# Correction: ‘They eat it like sweets’: A mixed methods study of antibiotic perceptions and their use among patients, prescribers and pharmacists in a district hospital in Kabul, Afghanistan

**DOI:** 10.1371/journal.pone.0299303

**Published:** 2024-02-15

**Authors:** Doris Burtscher, Rafael Van den Bergh, Masood Nasim, Gbane Mahama, Sokhieng Au, Anita Williams, Abdul Sattar, Suzanne Penfold, Catherine Van Overloop, Sahar Bajis

There are errors in the Funding and Competing Interests statements. The correct statements are:

**Funding:** Médecins sans Frontières (MSF) provided support in the form of salaries for all authors. The specific roles of each author are articulated in the ‘author contributions’ section. MSF programmatic funding covered all other costs associated with the study. The funder was involved in study design, data collection and analysis, decision to publish, and preparation of the manuscript.

**Competing Interests:** The authors have read the journal’s policy and have the following competing interests to declare: Médecins sans Frontières (MSF) provided support in the form of salaries for all authors. This does not alter our adherence to *PLOS ONE* policies on sharing data and materials. There are no patents, products in development or marketed products associated with this research to declare.
