# Supplementary material for: ‘They eat it like sweets’: A mixed methods study of antibiotic perceptions and their use among patients, prescribers and pharmacists in a district hospital in Kabul, Afghanistan
Source: PLoS One. 2021 Nov 19;16(11):e0260096. doi: 10.1371/journal.pone.0260096 (PMC8604360; doi:10.1371/journal.pone.0260096)
Supplement: S1 Appendix — (PDF) [file pone.0260096.s001.pdf]

Question guide patient and caretaker

Age, children, education, ethnic background, language, gender, occupation, province they come from, home many people live in the same household

Why did you come today to ASB hospital? Whom did you accompany today to ASB?

Health seeking behaviour

1. Where do you go for treatment first when you are sick?
2. Who or what decides where to go for treatment?
3. What influences your decision where to seek for treatment?

Drugs and medication

1. Where can you get drugs when you need them for some ill-health condition?
2. How far is (the hospital, the pharmacy, the drug store)?
3. Who usually goes for the drugs?
4. Can you get every medicine you like to have?
5. What are the difficulties to get drugs?
6. Which kind of drugs do you know?
7. (pills, capsules, serum (IV), injections, powder syrup, …)
8. Which kind of drugs is the strongest/fastest to cure?
9. Which kind of drug do you prefer?
10. What is the effect of this drug on the body?

Antibiotics

1. What is your opinion about AB? What does an AB mean to you?
2. AB = dirty drying pills, dirty cleaning pills, dryer of dirty things, orange pills, circled medicine, white tablet with line in the middle, powder syrup, zedi microbe, etc.
3. Which kind of words do people use to describe an AB?
4. What are the characteristics of AB?
5. What is the power of AB?
6. How much do you believe in AB?
7. For which kind of disease do you use AB?
8. What can an AB do to your body?
9. Do you know an AB (name)?
10. How and where can you get AB?
11. Do people use many times AB? Why?
12. Why do people like AB?
13. How do people know how to use the AB?
14. To whom can they ask?
15. How do people follow the instructions form the Dr or pharmacist?
16. What is the reason when they do not follow the advice of the Dr?
17. How do people trust the Dr or pharmacist?
18. Whom do they trust more?
19. Are there any dangers in taking AB?
20. Did you ever have the experience that the AB was not effective? Resistance? Please describe.
21. What could be the reason for that? What can be the consequence?
22. How could we prevent that?

Doctor patient relationship

1. Can you describe a good doctor?
2. What do you do when the doctor does not prescribe what you want/expect?

Doctors and Prescribers

Age, children, education, ethnic background, language, gender, occupation, province they come from, home many people live in the same household

Health seeking behaviour

1. Where do people go for treatment first?
2. Who or what decides where to go for treatment?
3. What influences their decision where to seek for treatment?

AB

1. How is the usage of AB among the people here?
2. What do they think about the AB?
3. Where do they buy AB?
4. Which AB are ‘easily’ available for them?
5. For which ones do they ask most?
6. Who in the family takes the decision which medication to take?
7. Which barriers do people face in coming to the health facility?
8. Which individuals especially?

Knowledge about AB

1. What do you tell patients about the AB?
2. What is the purpose of an AB?
3. What are the risks to take AB?
4. When is it not appropriate to take AB?
5. What are the benefits of AB?
6. How can you get AB resistance?
7. What are the causes of AB resistance?
8. How can AB resistance be prevented?
9. What do you think about the amount of AB the people use?
10. How do patients follow your instructions on taking AB?
11. Do they always finish the AB you prescribe them? Please explain
12. What do they do with the remaining AB?

Prescribing characteristics

1. How do you feel about prescribing AB according to the guidelines?
2. What are the difficulties you can face?
3. What are the problems you face with demands from patients and staff?
4. How do you deal with the expectations of patients and staff here?
5. For which illness do you prescribe AB?
6. How do you decide which AB you prescribe?
7. Which factors influence your decision on prescribing AB?
8. Which information do you give to your patients for the AB prescription?
9. How do patients react to your explanation?
10. How do you think patients follow your instructions?
11. What are the favourite types of AB for patients?
