# Supplementary material for: ‘They eat it like sweets’: A mixed methods study of antibiotic perceptions and their use among patients, prescribers and pharmacists in a district hospital in Kabul, Afghanistan
Source: PLoS One. 2021 Nov 19;16(11):e0260096. doi: 10.1371/journal.pone.0260096 (PMC8604360; doi:10.1371/journal.pone.0260096)
Supplement: S2 Appendix — (PDF) [file pone.0260096.s002.pdf]

QUESTIONNAIRE ON THE PERCEPTION, KNOWLEDGE AND USE OF ANTIBIOTICS
دانتی بیوتیکو د درک، پوهی او استعمال په هکله پوښتن پاڼه

Date/تاریخ ....

Name interviewerدمصاحبه کونکی نوم/: ....

Number of interview/دمصاحبو تعداد :....................

Placeځای/:
☐ Female OPD1 (triage) ښځینه OPD1(ترایاژ)
☐ Female OPD2 (consultations) ښځینه OPD2مشوره ګاني
☐ EPI (vaccination) EPI(واکسین)
☐ Male OPD دنارینه OPD
☐ Male caretaker area دنارینه پایوازانو ساحه
☐ Dispensary درملتون

PROFILE INTERVIEWEE/**دمصاحبه شوی شخص پروفایل**

Age of intervieweeدمصاحبه شوی شخص سن/: ....

Profile of the Intervieweeدمصاحبه شوی شخص پروفایل/ :
☐ Adult Patientکاهل ناروغ/
☐ Caretaker of child patient : *Specify age of child patient:*دماشوم ناروغ پایواز:د ناروغ سن مشخص کړی/
....

Genderجنس :☐ Femaleمونث☐ Male مذکر

Marital statusمدنی حالت:☐ Married متاهل☐ Single مجرد☐ Widowکونده

Area of residenceداوسیدنی ځای:☐ Urban ښار☐Ruralکلی

Language (mother tongue) (مورنی ژبه) ژبه: ☐ Pashtoپښتو☐ Dari دری☐other نور : ....

Educationتحصیلات:
☐ Illiterate بی سواد
☐ Primary school (1 to 9 grade) (اول صنف نه ترنهمه پوری) ابتدایه ښوونځی
☐ Secondary school (9 to 12 grade) (ثانوی ښوونځی) نهم صنف نه تردولسمه پوری
☐ Universityپوهنتون

Occupationدنده:
☐ No job بی کار
☐ Daily worker: *specify*روزمزد کارګر: مشخص کړی:.......................
☐ Job: *specify*دنده: مشخص کړی:

# Childrenداولادونوتعداد: ....

household members دکورنی غړی تعداد: ....

---

1. For which condition did you come (yourself or the person you came with)?
د څه لپاره تا سو دلته راغلی یاست (تاسو او یا هغه څوک چه تاسوسره راغلی ده؟

Open question (don't read the answers) - Possible to give more than 1 answer
آزاد پوښتنه (هیله کوو ځوابونه مه لولی) – امکان لری چه څوځوابونه هم ورکړی

☐ Fever / **تبه**
☐ Sore throat / **ستونی درد**
☐ Cough/breathing problem/ **ټوخی/ تنفسی مشکلات**
☐ Diarrhoea /
☐ (General) body pain /**(عمومی)دربدن درد**
☐ Infection / **انتان**
☐ Skin problems /**دپوستکی مشکلات**
☐ Diabetes / **شکر**
☐ Hypertension/**دوینی لوړفشار**
☐ Vaccination of my child / **دماشوم واکسین**
☐Other: /**نور**..

2. What did you do before coming to ASB to treat this condition?
مخ کی له دی چه احمدشاه بابا روغتون ته راشی، دخپل مشکل لپاره څه کوی؟

(Not relevant if coming for vaccination only)**(کوم کسان چه یوازې د واکسین لپاره راځی هغوی ته مربوط نه ده)**

Open question (don't read the answers) - Possible to give more than 1 answer
آزاد پوښتنه (هیله کوو ځوابونه مه لولی) – امکان لری چه څوځوابونه هم ورکړی

☐ We came directly to the Hospital / **مستقیم روغتون ته راغلو**
☐ We treated the condition at home/**تداوی موکورکی شروع کړی دی**
☐ We went to the drug store/local pharmacy/market / **درملتون ته/ مارکیت ته/ لاړو**
☐ We went to a private clinic/doctor / **شخصی ډاکتر/کلینیک ته لاړو**
☐ We consulted a Mullah/traditional healer/ **د ملا اویا محلی تداوی کونکی سره مشوره درلود**
☐ Other: **نور**..

3. Where do you usually get your medication?
تاسو معمولا خپل دارو د کوم ځای څخه لاس ته راوړی؟

Open question (don't read the answers) - Possible to give more than 1 answer
آزاد پوښتنه (هیله کوو ځوابونه مه لولی) – امکان لری چه څوځوابونه هم ورکړی

☐ Government pharmacy / **دولتی درملتون**
☐ Private pharmacy (Local drug store) / **شخصی درملتون(محلی دوکانونه)**
☐ Private pharmacy in private Clinic / **دشخصی کلینیک شخصی درملتون**
☐ Market (Bazaar) / **مارکیت(بازار)**
☐ In the street (black market)/ **له سړک غاړی نه (توربازار)**
☐ From relatives/ **دخپلوانوڅخه**
☐ Other:**نور**..

4. Which different types of medication do you know?
د درملونو څو نوعه ډولونه تاسی پیژنی؟

Open question (don't read the answers) - Possible to give more than 1 answer
آزاد پوښتنه (هیله کوو ځوابونه مه لولی) – امکان لری چه څوځوابونه هم ورکړی

☐ Injection / **پیچکاری**

☐ Pills / گولی

☐ Capsules /کپسول
☐ Serum /سیرم
☐ Syrup /شربت
☐ Powder syrup /پوډری شربت

☐ Antibiotics (Zedi microbe, Zedicherk, orange pills,)/ انتی بیتوتیک (درمیکروب ضد درمل, دچرک ضد درمل, نارنجی دنګه درمل ,...)

☐ Ointment / cream / کریم/ مرهم
☐ I don’t know / نه پوهیږم

☐ Other:نور...

5. What type of drug do you expect to receive during your visit here?
څه توقع لرى، کله چه دلته معاینى لپاره راځى نو کوم نوع درمل تاسوته درکړل شى؟

Open question (don’t read the answers) - Possible to give more than 1 answer
آزاد پوښتنه (هیله کوو ځوابونه مه لولی) – امکان لرى چه ځوځوابونه هم ورکړی

☐ Injection /پیچکاری
☐ Pills /گولی

☐ Capsules /کپسول
☐ Serum /سیرم
☐ Syrup /شربت
☐ Powder syrup /پوډری شربت

☐ Antibiotics (Zedi microbe, Zedicherk, orange pills...)/ انتی بیتوتیک (درمیکروب ضد درمل, دچرک ضد درمل, نارنجی دنګه درمل ,...)

☐ Ointment / cream /مرهم / کریم
☐ The doctor knows and decides /ډاکترپوهیږی او هغه تصمیم نیسی

☐ I don’t know / نه پوهیږم

☐ Other:نور...

6. What would you do if you don't receive the drug you expected to receive?
کله چه تاسو دخپل ضرورت وړ درمل ترلاسه نکړی، نوپه دی حالت کی تاسو څه کوی؟
Open question (don’t read the answers) - Possible to give more than 1 answer
آزاد پوښتنه (هیله کوو ځوابونه مه لولی) – امکان لری چه ځوځوابونه هم ورکړی

☐ Ask the doctor for it /له ډاکټرنه غواړم

☐ Ask the dispenser for it /له توضیح کونکی نه غواړم

☐ Go to another private clinic/doctor /بل شخصی کلینیک/ډاکټر ته ځم

☐ Go to a private pharmacy (drug store) /شخصی درملتون ته خم (دوکان ته)
☐ Go to the market (bazaar) /مارکیټ ته ځم (بازارته)

☐ Go to the streets (black market) /له سړک غاړی نه لاس ته راوړم (توربازار)

☐ Accept because the doctor knows and decides /ځه چه ډاکټروایی اویا تصمیم نیسی هغه منم

☐ I don’t know /نه پوهیږم

☐ Other:نور...

7. Do you agree with the following statements?
دلاندی جملو سره موافق یاست؟

Read each statement to the interviewee and indicate the answer for each statement
هرجمله مصاحبه شوی شخص ته ولولی او وګوری چه ځواب کوم جمله ته دلالت کوی

- Big number of pills/capsules can cure faster ☐yesهو☐ No نه
یوزیات شمیر ګولی/ کپسولونه کولای شی ډیر ژر دتداوی لامل وګرځی.

- Injections or serum can cure faster than pills ☐ yesهو☐ No نه
- پیچکاری اویا سیروم د ګولی په پرتله ډیر ژر د تداوی لامل ګرځی.

-**The quality of the pill is more important than the number of pill** ☐ **yesهو** ☐ **Noنه**
د ګولیو کیفیت د ګولیو د تعداد په پرتله ډیر مهم ده

-**Taking different pills together will cure you faster than taking the same ones** ☐ **yesهو** ☐ **Noنه**
د ګولیو مختلف ډولونه د یو ډول ګولی په پرتله ډیر ژر د تداوی لامل ګرځی

8. Who explains to you how to use the drug?
تاسو د کوم شخص په مشوره له درملو څخه ګټه پورته کوی؟

Open question (don't read the answers) - Possible to give more than 1 answer
آزاد پوښتنه (هیله کوو ځوابونه مه لولی) – امکان لری چه څوځوابونه هم ورکړی

☐ Doctor /**ډاکټر**

☐ Dispenser/pharmacist/ **فارمسست/توضیح کونکی**

☐ Follow what is written on the packet by the pharmacist **دهغه معلومات برېنسټ چه فارمسست د درمل د پاکټ په شا کی**

☐ Relative/Family / **خپلوان/کورنی**
☐ Someone who can read and write /**یوچا چه سواد ولری**
☐ I don't know / **نه پوهیږم**

☐ Other:**نور**..

9. Are the doctors/pharmacist instructions always clear?
آیا د ډاکتران او فارمسستان معلومات همیشه صحیح ده؟

☐ **Yesهو**
☐ **Noنه**

10. What does the doctors/prescribers explain to you on how to use the medication?
د درملو د استعمال په هکله ډاکتران/ مشوره ورکونکی تاسو ته څنګه معلومات ورکوی؟
Read each statement to the interviewee and indicate the answer for each statement
هرجمله مصاحبه شوی شخص ته ولولی او وګوری چه ځواب کوم جمله ته دلالت کوی

- **Number of pills per day** ☐ **yesهو** ☐ **Noنه**
د ګولیو تعداد په ورځ کی
- **Number of times per day** ☐ **yesهو** ☐ **Noنه**
د درمل خوړل وخت په ورځ کی
- **Time(s) of the day to take the drug** ☐ **yesهو** ☐ **Noنه**
د درمل خوړل دفعات په ورځ کی
- **Total number of days (for how long)** ☐ **yesهو** ☐ **Noنه**
ترڅو ورځو پوری درمل ونیول شی
- **Need to finish all the drugs** ☐ **yesهو** ☐ **Noنه**
ټول درمل باید وخوړل شی
- **Need to take the drug with food** ☐ **yesهو** ☐ **Noنه**
درمل باید د ډوډی سره واخیستل شی

11. Have you heard about "Antibiotics"? (Zedimicrobe, Zedicherk...)
آیا تاسو د انتی بیوتیک په باره کی کوم څه اوریدلی یاست؟ (د میکروب ضد، د چرک ضد)

☐ **Yes/ هو**
☐ **No/ نه**

12. What do you use "antibiotics" for ?
تاسو انتی بیوتیک په کومو مواردو کی استعفاده کوی؟

(explain AB with local terms for example dirty dryer medicine, orange pills, powder syrup,...)
(انتی بیوتیک د عوام په ژبه خلک ته تشریح ګړی، دمثال په توګه: د چرک وچ کونکی درمل، نارنجی درمل، پوډری شربت....)
Open question (don't read the answers) - Possible to give more than 1 answer
آزاد پوښتنه (هیله کوو ځوابونه مه لولی) – امکان لری چه څوځوابونه هم ورکړی

☐ Pain/ **درد**
☐ illness(es)/disease(s)/**بیماری/امراض**

☐ Infection/ انتان
☐ To kill/dry microbes/وژل/میکروب وچ کول
☐ Fever/ تبه
☐ Sore throat/ ستونی درد
☐ Cough/breathing problem/ تنفسی مشکلات/سرفه
☐ Diarrhoea/ نس ناستی
☐ (General) body pain/ دبدن درد (عمومی)
☐ Skin problems/ دپوستکی مشکلات
☐ Diabetes/ شکر
☐ Hypertension/ دوینی لوړ فشار
☐ After delivery/ د ولادت نه وروسته
☐ I don't know/ نه پوهیږم
☐ Otherنور :...

13. Do you agree with the following statements about antibiotics?
تاسی د لاندنی جملو سره د انتی بیوتیک په تړاو موافق یاست؟

Read each statement to the interviewee and indicate the answer for each statement
هرجمله مصاحبه شوی شخص ته ولولی او وګوری چه ځواب کوم جمله ته دلالت کوی

- Antibiotics can cure all the conditions ☐ yesهو☐ Noنه
انتی بیوتیک کولای شی ټول حالتونه تداوی کړی
- Antibiotics are useful to kill microbes ☐ yesهو☐ Noنه
انتی بیوتیک درمیکروبونو په وژل کی ډیرمهم ده
- Antibiotics are the best drugs to cure quickly ☐ yesهو☐ Noنه
انتی بیوتیک درمل د عالی درملوپه جمله کی راځی او ډیرچټک د تداوی سبب ګرځی
- Antibiotics is a good medication to treat infection ☐ yesهو☐ Noنه
انتی بیوتیک یو ډیرښه درمل درانتاناتو د له منځه وړل په برخه کی ده
- Antibiotics can be used to treat pain ☐ yesهو☐ Noنه
انتی بیوتیکونه د درد په له منځه وړلو کی ډیرموثردی.
- Antibiotics are only for children ☐ yesهو☐ Noنه
انتی بیوتیک درمل یواځی د ماشومانو لپاره ده.

14. Do you sometimes stop taking the prescribed antibiotics before you finish all of it?
آیا داسی کیدای شی چه تاسو خپل انتی بیوتیک استعمال، مخ کی له دی چی ټول ختم شی قطع کړی؟
☐ Yes /هو
☐ No /نه

15. If yes, what is the reason to stop?
که چیری ځواب مو هو وی، نو دلیل یی څه ده؟

Open question (don't read the answers) - Possible to give more than 1 answer
آزاد پوښتنه (هیله کوو ځوابونه مه لولی) – امکان لری چه ځوځوابونه هم ورکړی

☐ Feeling better/cured /درملنه وشو/ښه احساس کوم
☐ The drug does not work/ ددرمل دوزموثرنه وو
☐ Bad smell/taste of the drug/ بد بوی لری/د درمل خوند
☐ Getting more sick (side-effects) / نارویغږملاهم
☐ The drug got lostدرمل می ورک شو
☐ Other:نور...

16. What do you do with the remaining prescribed antibiotics?
له باقی انتی بیوتیکونو سره څه کوی؟

Open question (don't read the answers) - Possible to give more than 1 answer
آزاد پوښتنه (هیله کوو ځوابونه مه لولی) – امکان لری چه ځوځوابونه هم ورکړی

☐ We keep it at home to use another time /کورکی ساتواوکیدای شی چه بل کوم وخت ورځینی استفاده وکو

☐ We throw it away/ ایسته اچوو
☐ We give it to relatives/ خپل دوستانوته ورکوو
☐ Other:نور...

17. Do you have "antibiotic" at home ?
آیا تاسو کورکی کوم انتی بیوتیک لری؟

(explain AB with local terms for example dirty dryer medicine, orange pills, powder syrup,...)
(انتی بیوتیک د عوام په ژبه خلک ته تشریح کړی، دمثال په توګه: د چرک وچ کونکی درمل، نارنجی درمل، پوډری شربت....)

☐ Yes /هو
☐ No /نه

18. If yes, where do they come from?
که ځواب هو وی، د کوم ځای څخه لاس ته راوړی؟

Open question (don't read the answers) - Possible to give more than 1 answer
آزاد پوښتنه (هیله کوو ځوابونه مه لولی) – امکان لری چه څوځوابونه هم ورکـړی

☐ Remaining AB from former treatment / له پخوانی درملنی نه پاتی شوی انتی بیوتیک
☐ Friends and Family / دوستان اوکورنی
☐ Pharmacy / drug store / دوکان /درملتون
☐ We bought them / راوړوخپلهمونږپه

☐ Other:نور...

19. If yes, how do you use them?
که ځواب هو وی، څنګه یی استعمالوی؟

Read each question to the interviewee and indicate the answer for each question
هرجمله مصاحبه شوی شخص ته ولولی او وګوری چه ځواب کوم جمله ته دلالت کوی

**- Do you use it for the same condition?**<br>**آیا تاسو دغه انتی بیوتیک د مشابه حالت په درملنه کی استفاده کوی؟**	**☐Yesهو☐ Noنه**
**- Do you use it for other conditions?**<br>**آیا تاسو دغه انتی بیوتیک د مختلف حالت په درملنه کی استفاده کوی؟**	**☐Yesهو☐ Noنه**
**- Do you use it for the same person it was prescribed for?**<br>**آیا هغه شخص باندی تطبیق کوی چه ورته تجویز شوی وو؟**	**☐Yesهو☐ Noنه**
**- Do you use it for other sick family members?**<br>**آیا د کورنی بل غړی ته تطبیق کوی؟**	**☐Yesهو☐ Noنه**
**- If the drug was prescribed for a child, can you use it for an adult?**<br>**که چیری دغه درمل یو ماشوم ته تجویز شوی وی، نو تاسوکولای شی چه یو کاهل ته هم ورکړی؟**	**☐Yesهو☐ Noنه**
**- If the drug was prescribed for an adult, can you use it for a child?**<br>**که چیری دغه درمل یوکاهل ته تجویز شوی وی، نو تاسوکولای شی چه یو ماشوم ته هم ورکړی؟**	**☐Yesهو☐ Noنه**
