# Supplementary material for: ‘They eat it like sweets’: A mixed methods study of antibiotic perceptions and their use among patients, prescribers and pharmacists in a district hospital in Kabul, Afghanistan
Source: PLoS One. 2021 Nov 19;16(11):e0260096. doi: 10.1371/journal.pone.0260096 (PMC8604360; doi:10.1371/journal.pone.0260096)
Supplement: S1 Table — (DOCX) [file pone.0260096.s003.docx]

**S1 Table. Responses to Survey Questions by Patients and Caretakers at ASB District Hospital, Kabul, Afghanistan, between October 10 and November 11, 2014**

|  | Yes | | | No | |
| --- | --- | --- | --- | --- | --- |
|  | n | | % | n | % |
| 1. For which condition did you come? |  | |  |  |  |
| Fever | 52 | | 15% | 299 | 85% |
| Sore Throat | 40 | | 11% | 311 | 89% |
| Cough/Breathing problems | 37 | | 11% | 314 | 89% |
| Diarrhoea | 16 | | 5% | 335 | 95% |
| General body pain | 100 | | 28% | 251 | 72% |
| Infection | 3 | | 1% | 348 | 99% |
| Skin infection | 20 | | 6% | 331 | 94% |
| Diabetes | 24 | | 7% | 327 | 93% |
| Hypertension | 25 | | 7% | 326 | 93% |
| Vaccination of child | 28 | | 8% | 323 | 92% |
| UTI/STI | 22 | | 6% | 329 | 94% |
| Obstetrics/Gynaecological | 21 | | 6% | 330 | 94% |
| Gastrointestinal | 30 | | 9% | 321 | 91% |
| Other | 70 | | 20% | 289 | 82% |
| 2. What did you do before coming to ASB? |  | |  |  |  |
| Direct to Hospital | 240 | | 68% | 111 | 32% |
| Treated at Home | 4 | | 1% | 347 | 99% |
| Drug store/Pharmacy | 34 | | 10% | 317 | 90% |
| Private Clinic/Doctor | 79 | | 23% | 272 | 77% |
| Mullah/Traditional | 5 | | 1% | 346 | 99% |
| Other | 5 | | 1% | 346 | 99% |
| 3. Where do you usually get your medication? |  | |  |  |  |
| Government Pharmacy | 149 | | 42% | 202 | 58% |
| Private Pharmacy/Drug store | 218 | | 62% | 133 | 38% |
| Private Pharmacy/Clinic | 125 | | 36% | 226 | 64% |
| Any Private Pharmacy | 305 | | 87% | 46 | 13% |
| Market/Street | 6 | | 2% | 345 | 98% |
| MSF | 11 | | 3% | 340 | 97% |
| Other | 2 | | 1% | 349 | 99% |
| 4. Which different types of medication do you know? |  | |  |  |  |
| Injection | 195 | | 56% | 156 | 44% |
| Pills | 331 | | 94% | 20 | 6% |
| Capsules | 174 | | 50% | 177 | 50% |
| Serum (intravenous line) | 154 | | 44% | 197 | 56% |
| Syrup | 258 | | 74% | 93 | 26% |
| Powdered Syrup | 59 | | 17% | 292 | 83% |
| Antibiotics | 39 | | 11% | 312 | 89% |
| Ointment/Cream | 66 | | 19% | 285 | 81% |
| Don't know | 6 | | 2% | 345 | 98% |
| Other | 10 | | 3% | 341 | 97% |
| 5. What type of drug do you expect to receive? |  | |  |  |  |
| Injection | 18 | | 5% | 333 | 95% |
| Pills | 51 | | 15% | 300 | 85% |
| Capsules | 14 | | 4% | 337 | 96% |
| Serum (intravenous line) | 16 | | 5% | 335 | 95% |
| Syrup | 44 | | 13% | 307 | 87% |
| Powdered Syrup | 4 | | 1% | 347 | 99% |
| Antibiotics | 4 | | 1% | 347 | 99% |
| Ointment/Cream | 3 | | 1% | 348 | 99% |
| Doctor decides | 266 | | 76% | 85 | 24% |
| Don't know | 2 | | 1% | 349 | 99% |
| Other | 12 | | 3% | 339 | 97% |
| 6. What would you do if you don't receive the drugs you expect? | | | | | |
| Ask Doctor | 11 | | 3% | 340 | 97% |
| Ask Dispenser | 2 | | 1% | 349 | 99% |
| Go to private clinic | 105 | | 30% | 246 | 70% |
| Go to private pharmacy | 40 | | 11% | 311 | 89% |
| Go to market | 3 | | 1% | 348 | 99% |
| Go to street/black market |  | | 0% | 351 | 100% |
| Accept doctor's decision | 179 | | 51% | 172 | 49% |
| Don't know | 3 | | 1% | 348 | 99% |
| Complain | 15 | | 4% | 336 | 96% |
| Go somewhere else | 141 | | 40% | 210 | 60% |
| 7. Do you agree with: |  | |  |  |  |
| More pills cure faster | 174 | | 50% | 164 | 47% |
| Injections/serum (intravenous line) cures faster | 296 | | 84% | 48 | 14% |
| Quality is more important than quantity | 342 | | 97% | 5 | 4% |
| Different pills together cure faster | 182 | | 52% | 161 | 46% |
| 8. Who explains how to use the drug? |  | |  |  |  |
| Doctor | 301 | | 86% | 50 | 14% |
| Dispenser | 253 | | 72% | 98 | 28% |
| Written on packet (pharmacist) | 15 | | 4% | 336 | 96% |
| Relative | 89 | | 25% | 262 | 75% |
| Someone literate | 43 | | 12% | 308 | 88% |
| Don't know | 2 | | 1% | 349 | 99% |
| Other | 23 | | 7% | 328 | 93% |
| 9. Are the instructions always clear? | 331 | | 94% | 16 | 5% |
| 10. What do the doctors/prescribers explain to you? |  | |  |  |  |
| Number of pills/day | 339 | | 97% | 11 | 3% |
| Number of times/day | 345 | | 98% | 5 | 1% |
| Times to take drug | 341 | | 97% | 7 | 2% |
| Duration | 311 | | 89% | 35 | 10% |
| Need to finish | 305 | | 87% | 44 | 13% |
| Need to take with food | 291 | | 83% | 56 | 16% |
| 11. Have you heard of antibiotics? | 228 | | 65% | 115 | 33% |
| 12. What do you use antibiotics for? |  | |  |  |  |
| Pain | 69 | | 20% | 282 | 80% |
| Illnesses | 16 | | 5% | 335 | 95% |
| Infections | 43 | | 12% | 308 | 88% |
| Kill/dry microbes | 111 | | 32% | 240 | 68% |
| Fever | 18 | | 5% | 333 | 95% |
| Sore throat | 52 | | 15% | 299 | 85% |
| Cough/breathing problem | 72 | | 21% | 279 | 79% |
| Diarrhoea | 19 | | 5% | 332 | 95% |
| General body pain | 44 | | 13% | 307 | 87% |
| Skin infection | 10 | | 3% | 341 | 97% |
| Diabetes | 0 | | 0% | 351 | 100% |
| Hypertension | 2 | | 1% | 349 | 99% |
| After delivery | 4 | | 1% | 347 | 99% |
| Don't know | 102 | | 29% | 249 | 71% |
| RTI | 50 | | 14% | 301 | 86% |
| Flu | 20 | | 6% | 331 | 94% |
| Blood/Bleeding | 8 | | 2% | 343 | 98% |
| Malaria | 6 | | 2% | 345 | 98% |
| 13. Do you agree with the following about antibiotics? |  |  | |  |  |
| Antibiotics can cure all conditions | 62 | 18% | | 237 | 68% |
| Antibiotics can kill microbes | 225 | 64% | | 76 | 22% |
| Antibiotics can cure illness quickly | 198 | 56% | | 96 | 27% |
| Antibiotics are the best for infection | 208 | 59% | | 91 | 26% |
| Antibiotics can treat pain | 118 | 34% | | 188 | 54% |
| Antibiotics are only for children | 8 | 2% | | 300 | 85% |
| 14. Do you sometimes stop taking the antibiotics before finishing? | | | | | |
|  | 82 | 23% | | 240 | 68% |
| 15. If so, reasons why (multiple options allowed): |  |  | |  |  |
| Feeling better/cured | 70 | 85% | | 12 | 15% |
| Drug does not work | 1 | 1% | | 81 | 99% |
| Bad smell/taste of the drug | 5 | 6% | | 77 | 94% |
| Side effects | 11 | 13% | | 71 | 87% |
| Drugs got lost |  |  | | 82 | 100% |
| 16. If so, what they did with remaining drugs (multiple options allowed): | | | | | |
| Kept for future use | 37 | 45% | | 45 | 55% |
| Threw them away | 46 | 56% | | 36 | 44% |
| Gave to relatives | 2 | 2% | | 80 | 98% |
| 17. Do you have antibiotics at home? | 122 | 35% | | 229 | 65% |
| 18. If have antibiotics at home, where obtained: |  |  | |  |  |
| Remaining from former treatment | 108 | 89% | | 14 | 11% |
| Pharmacy/drug store purchase | 81 | 66% | | 41 | 34% |
| Friends and family | 27 | 22% | | 95 | 78% |
| Purchased on black market | 24 | 20% | | 98 | 80% |
| From health facility | 4 | 3% | | 118 | 97% |
| 19. If have antibiotics at home, agreement with the following statements: | | | | | |
| Used for the condition for which it was prescribed | 104 | 85% | | 18 | 15% |
| Used by the person for whom is was prescribed | 80 | 66% | | 42 | 34% |
| Used for other conditions other than the one prescribed | 26 | 21% | | 96 | 79% |
| Used for other sick family members | 28 | 23% | | 94 | 77% |
| If the drug was prescribed for a child,  you can use it for an adult | 4 | 3% | | 118 | 97% |
| If the drug was prescribed for an adult,  you can use it for a child | 4 | 3% | | 118 | 97% |
