# Supplementary material for: ‘They eat it like sweets’: A mixed methods study of antibiotic perceptions and their use among patients, prescribers and pharmacists in a district hospital in Kabul, Afghanistan
Source: PLoS One. 2021 Nov 19;16(11):e0260096. doi: 10.1371/journal.pone.0260096 (PMC8604360; doi:10.1371/journal.pone.0260096)
Supplement: Attachment — Submitted filename: Response to reviewers .docx [file pone.0260096.s005.docx]

**Response to academic editor and reviewer(s)**

*Thank you very much for considering a revised manuscript. We have addressed the reviewer comments and made revisions to the manuscript. Please see below our response to the reviewer comments in italics (with page numbers pertaining to tracked-manuscript).*

Journal Requirements:

When submitting your revision, we need you to address these additional requirements.

1. Please ensure that your manuscript meets PLOS ONE's style requirements, including those for file naming. The PLOS ONE style templates can be found at

https://journals.plos.org/plosone/s/file?id=wjVg/PLOSOne_formatting_sample_main_body.pdf and

https://journals.plos.org/plosone/s/file?id=ba62/PLOSOne_formatting_sample_title_authors_affiliations.pdf

*Thank you for this recommendation, we have reconsulted the journal’s formatting guidelines and have changed the manuscript accordingly.*

2. Please include additional information regarding the survey or questionnaire used in the study and ensure that you have provided sufficient details that others could replicate the analyses. For instance, if you developed a questionnaire as part of this study and it is not under a copyright more restrictive than CC-BY, please include a copy, in both the original language and English, as Supporting Information.

*We will submit the questionnaire used in the quantitative part of the study as additional information. The questionnaire is one document in Pashto and English language. Additionally we will submit the qualitative question guide. We will also upload the survey data as a supplementary file.*

3. Upon re-submitting your revised manuscript, please upload your study’s minimal underlying data set as either Supporting Information files or to a stable, public repository and include the relevant URLs, DOIs, or accession numbers within your revised cover letter. For a list of acceptable repositories, please see http://journals.plos.org/plosone/s/data-availability#loc-recommended-repositories. Any potentially identifying patient information must be fully anonymized.

Important: If there are ethical or legal restrictions to sharing your data publicly, please explain these restrictions in detail. Please see our guidelines for more information on what we consider unacceptable restrictions to publicly sharing data:

http://journals.plos.org/plosone/s/data-availability#loc-unacceptable-data-access-restrictions. Note that it is not acceptable for the authors to be the sole named individuals responsible for ensuring data access.

We will update your Data Availability statement to reflect the information you provide in your cover letter.

*We have provided the quantitative data as a supplementary file. The qualitative data cannot be shared due to the following ethical reasons:*

- *Even though de-identified, transcripts came from a vulnerable and restricted group of respondents and the narratives there contained might disclose personal aspects of their life. In this we follow our ERB protocol for protecting participants*
- *In the Informed consent form we did not indicate the possibility for public access to their narratives; it was only stated that these will be used for analysis only by the research group – again following ERB protocol and that quotes will be used anonymized in a publication.*

4. A factor of concern to me is the fact that there may be only two Afghans as co-authors in this manuscript. Is there any reason for this lesser number? I am not aware of the involvement of Afghans in their countries healthcare system and I also know that MSF may depend more on volunteer doctors.

*Thanks for this important comment. We agree with the reviewer that there is an underrepresentation of Afghan co-investigators in this study, however; our two Afghan colleagues and co-authors on this manuscript have made substantial contributions to the design, implementation of the study and interpretations of findings. In addition, an Afghan pharmacy assistant was trained to conduct data entry for the study as part of research skills building in the hospital. MSF continues to take measures to support and provide training to local staff to lead research projects. It should be noted that MSF employs local doctors in accordance with local requirements – they are not volunteers.*

5. The study was conducted in 2014 and there is a long gap between the conduct of the study and the submission of the manuscript in 2021. The difficult situation in Afghanistan may have contributed though I am not sure.

*While there has been an extended period of time between the conduct of this study and submission of manuscript, the nature of our findings remains representative of the community, relevant and applicable to the current context and issue of antimicrobial use and resistance and has important policy and practice implications.*

6. There is a lack of clear description of how the findings from the qualitative study were used to develop the questionnaire. There is no clear demarcation between the qualitative and quantitative phase of the study.

*We have added text in the “Quantitative component” of the methods section outlining how the findings from the qualitative phase was used to develop the survey. We have also added time periods for when the two phases were conducted in order to delineate between the two phases of the study. Please see page 4 and page 7.*

7. The authors have not mentioned how the data from the questionnaire was analysed.

*We have provided further detail in the methods section to clarify how this analysis was conducted. Please refer to page 8 in the manuscript.*

8. What exactly is meant by a health promoter?

*The role of health promoters is to facilitate and deliver health messages and education to patients/communities around disease prevention and control. They play an important role in ensuring health services are accessible and relevant to communities. In the Ahmed Shah Baba hospital in Kabul health promotion activities included providing information to caretakers in the male and female waiting area on antibiotic characteristics, and antibiotic resistance, and for which ill-health conditions antibiotics should be used. Additionally, people were sensitised about mental health services provided by MSF.*

9. Are the medical staff Afghans or expatriates? My understanding on reading the manuscript is that the doctors working in the hospital also have their private practice. I am not sure if this is correct.

*At the time of the study, there were three international medical staff and 215 national medical staff employed by MSF at the ASB hospital. Additional to this there were 43 medical Ministry of Public Health (MoPH) staff supported by MSF working at the hospital. In accordance with local human resources regulations, national staff were entitled to practice in private clinics outside of their employment at the hospital.*

10. The findings of the study point to a disturbing situation which is like that seen in other deprived areas. The authors have described the strengths and limitations of the study clearly. Considering the limitations of the current study do the authors recommend further studies in other regions of the country, though, this may be difficult.

*Ideally, we would repeat the study in our other projects, and we are in discussions with the team in Afghanistan regarding the possibility of doing a similar study to update and extend our knowledge on this topic. That said, considering the current security context this idea may be delayed.*

11. In the supplementary file question 4, what is meant by serum?

*Thank you for this clarifying question. In the local context, serum means medication received through an intravenous line.*

12. This is an important study and I assume the situation may not have changed substantially in the seven years since the study was conducted.

*We agree with the reviewer that despite the length of time since the conduct of study, the findings are still relevant and important in addressing knowledge gaps and policy implications in Afghanistan and the regions more broadly.*

The following responses are from comments made in the pdf of our article

1. Are expired medicines also circulated?

*We are not aware of anything documented but this is possible; it would be outside of the scope of this paper to discuss this subject.*

2. This can be rewritten in a simple language.

*We have made changes to that sentence to clarify the language. Please see on page 3 in the manuscript.*

3. What was the basis for 30% prevalence?

*The 30% prevalence assumption was based upon findings from similar studies.*

4. How was the questionnaire validated?

*The data was double-entered into EpiData and cross-checked between the two entries. This is what was meant by validated. The manuscript has been changed to say “cross-checked” to clarify this. Additionally, text has been added in line 194 that the questionnaire was pre-piloted in the population. Please refer to page 8.*
