# Supplementary material for: ‘They eat it like sweets’: A mixed methods study of antibiotic perceptions and their use among patients, prescribers and pharmacists in a district hospital in Kabul, Afghanistan
Source: PLoS One. 2021 Nov 19;16(11):e0260096. doi: 10.1371/journal.pone.0260096 (PMC8604360; doi:10.1371/journal.pone.0260096)
Supplement: Attachment — Submitted filename: Response to Reviewers final.docx [file pone.0260096.s006.docx]

**Response to journal editor**

*Thank you very much for considering the revised manuscript. We have addressed the editor comments and made revisions to the manuscript. Please see below our response.*

1. Please include captions for your Supporting Information files at the end of your manuscript, and update any in-text citations to match accordingly. Please see our Supporting Information guidelines for more information: <http://journals.plos.org/plosone/s/supporting-information>.

We have included captions and updated in-text citations, please see line 216 and 509.

2. Thank you for letting us know that your data is confidential and cannot be publicly shared. According to PLOS policy, data that cannot be publicly shared should be made available on request through an ethics or data access committee to researchers who meet the criteria for access to confidential data.

*We have added data access committee with contact details to access data on request.*
